# Systematic review of the literature and meta-analysis of the long-term likelihood of contralateral total hip arthroplasty

**DOI:** 10.1530/EOR-2024-0026

**Published:** 2025-05-05

**Authors:** Jonas Liebe, Michel Schläppi, Christoph Meier, Peter Wahl

**Affiliations:** ^1^ Division of Orthopaedics and Traumatology, Cantonal Hospital Winterthur, Winterthur, Switzerland; ^2^ Faculty of Medicine, University of Berne, Berne, Switzerland

**Keywords:** meta-analysis, hip prosthesis, total hip arthroplasty, THA, contralateral

## Abstract

**Purpose:**

- Total hip arthroplasty (THA) is performed commonly for various end-stage diseases of the hip joint. However, the likelihood of a subsequent contralateral THA (cTHA) after primary unilateral index THA (iTHA) remains insufficiently defined, with reported rates of 13–29.1% after 5 years and 8.7–54% after 10 years of follow-up. This review aims to determine the long-term likelihood over time of cTHA after iTHA.

**Methods:**

- Systematic review of the literature with meta-analysis, including any clinical study published until July 2022, evaluating or documenting the likelihood over time of cTHA after iTHA, independently of the etiology. Excluded were cTHA within 12 months. A total of 21 studies, including 1,456,071 patients, who subsequently received 249,117 cTHA, were analyzed. Kaplan–Meier analysis was performed, weighting data on sample size, considering death as competing risk.

**Results:**

- At 5 years after iTHA, likelihood of cTHA was 17.8% (95% confidence interval 12.3–23.3%). At 10 years, this likelihood increased to 22.7% (16.1–29.4%), with a marginal subsequent increase. The likelihood increased slightly considering death as competing risk.

**Conclusion:**

- Nearly every fourth THA patient will require cTHA within 10 years. The likelihood of cTHA in this review appears to fall within the lower third of previously published ranges. However, most cTHA are required within the first years. Our findings suggest that the likelihood of requiring cTHA within the initial 10 years is approximately twice as high as the likelihood of requiring revision of the iTHA.

## Introduction

Total hip arthroplasty (THA) is a cost-effective and well-established intervention for various end-stage diseases of the hip joint (1, 2, 3, 4, 5). In addition to its impact on enhancing quality of life (QoL) and quality-adjusted life years, THA has even been shown to improve cognitive function in patients suffering from end-stage osteoarthritis (OA) (3, 4, 5, 6, 7). The lifetime likelihood of requiring THA for OA in high-income countries is reported to range from 8.7 to 15.9% for females and from 6.3 to 8.6% for males (8). This likelihood is expected to increase significantly in the future, particularly due to aging populations and increasing functional demands (8, 9, 10, 11).

We defined the first THA on one side as index total hip arthroplasty (iTHA), independent of the etiology. Any subsequent THA of the contralateral hip is referred to as contralateral THA (cTHA). The likelihood of requiring a subsequent cTHA after iTHA has not been assessed conclusively so far. The results of previous studies show great discrepancy between reported likelihoods, varying from 13 to 29.1% at 5 years after iTHA, respectively, from 8.7 to 54% at 10 years follow-up (12, 13, 14). There is, however, a wide range of inclusion criteria and differences in study design between publications reporting or assessing the likelihood of cTHA, limiting comparability and risk assessment. Variations in study size, design (retrospective vs prospective) and data extraction methods further contribute to the differing results. This may be exemplified by Amstutz *et al.*, who focused on patients with idiopathic osteoarthritis without contralateral symptoms, while Sayeed *et al.* analyzed patients aged 50–75 after Charnley THA (12, 13). Ritter *et al.* examined both unilateral and bilateral osteoarthritic hips, leading to broader variability in outcomes (14).

To the best of our knowledge, this is the first systematic review and meta-analysis investigating the likelihood for requirement of cTHA after iTHA. As health systems are exposed worldwide to growing demands and financial pressure, estimating the risk of future cTHA is crucial to improve healthcare planning, optimizing resource allocation and ensuring timely follow-up for patients (5, 8, 9, 10, 11, 15). The primary aim of this study was to quantify the likelihood of cTHA following iTHA, providing a foundation for future adjustments in healthcare strategies. This could result in more efficient scheduling of follow-up care, timely interventions and overall cost savings, while also enhancing patient outcomes and satisfaction.

## Methods

Studies were identified in two databases according to the AMSTAR guidelines (16), once in the Medline database from the National Library of Medicine (NLM) using the search engine PubMed and once in the Cochrane Library. Inclusion criteria were any clinical study, published until July 2022 inclusively, evaluating or documenting the likelihood over time of cTHA after primary, unilateral iTHA, independent of etiology. We excluded studies, respectively results, if cTHA was performed obviously within 12 months of iTHA. Furthermore, we excluded studies when the full text was unavailable, abstract-only publications, articles in press, conference abstracts, editorials, author responses or publications in books. Studies with data not reliably extractable, duplicate or overlapping data were also excluded.

**Figure 1:**
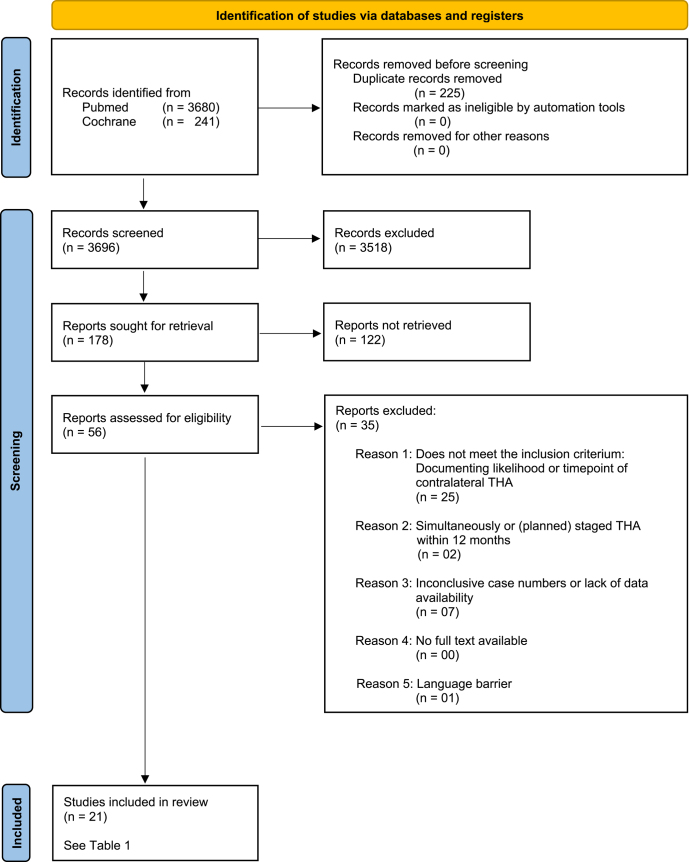
Flowchart showing the process of study identification and inclusion, respectively exclusion, using the PRISMA (preferred reporting items for systematic reviews and meta-analyses) synthesis methods.

Our search strategy followed the PRISMA 2020 guidelines for systematic reviews (see Fig. 1). The following Medical Subject Headings (MeSH) and terms were used in the search process (see Tables 1 and 2). To include any potentially relevant study, we applied a text word search (*tw*) with truncation of the key words (*). The language of the publications was limited to English, and in PubMed, the species filter ‘human’ was applied. The bibliographies of any relevant publications were also cross-checked for potentially eligible references. Screening of the potential studies was done and recorded by the first author without any automation tools. All records were collected into one EndNote library (version 20.4, Clarivate, USA) to manually delete duplicates. Both the first and the second author then reviewed the publications regarding inclusion and exclusion criteria. The first round was done by the first author, screening title and abstract only. The second round was done by both the first and the second author, applying inclusion/exclusion criteria to the full text of the publication. Reasons for exclusion are documented (Fig. 1). Inclusion and exclusion of a study was decided on a consensus basis between both evaluators.

If available, data were extracted from tables or figures (17). In some of the studies, data were automatically extracted from the Kaplan–Meier (KM) survivorship figures (18, 19) using the juicr package (version 0.1) within R statistical software (v4.2.1; R Core Team 2022).

**Figure 2:**
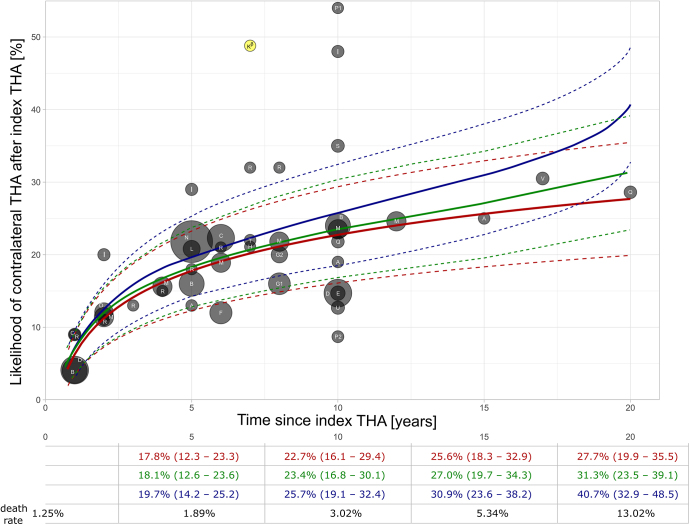
Kaplan–Meier analysis showing the likelihood of cTHA following iTHA over time as a percentage. The data were weighted according to the sample size of the studies included, illustrated by a dot size proportional to the number of patients. Kβ (yellow dot) represent data of patients suffering only from AVNFH. The red curve represents the likelihood of cTHA including death rate and the statistical assumption that a deceased patient would not undergo subsequent cTHA. This model represents the baseline with the average likelihood of cTHA, while the two other models incorporated additional factors. The green curve represents a scenario where patients would not die but continue to receive cTHA with the same likelihood as all survivors. The blue curve considers the likelihood of cTHA with censored death and the assumption that every deceased patient would have undergone subsequent cTHA. This model combines the baseline likelihood of the red curve, with addition of the percentage of deceased patients over time. This hypothetical scenario represents the upper limit of potential cTHA occurrence. To simulate elimination through the competing risk of death, the global death rate according to the World Health Organization was used, assuming the average patient age of 65–69 at iTHA. The dotted lines indicate the 95% confidence interval. G1: Australian Orthopaedic Arthroplasty Association National Joint Replacement Registry (AOA NJRR); G2: Norwegian Arthroplasty Register (NAR); Kβ: Primary diagnosis: Avascular necrosis of the femoral head (AVNFH); P1: Bilateral osteoarthritis (OA); P2: Unilateral osteoarthritis (OA).

Data weighting was performed based on the number of patients included in each study. KM analysis was performed, considering the likelihood of cTHA following iTHA over time as a percentage. To simulate elimination through the competing risk of death, the global death rate of industrialized countries according to the World Health Organization was used, assuming the average patient age of 65–69 at iTHA (‘world bank income group’ = HIGH INCOME). To assess the role of the death rate, the following models were applied (Fig. 2). The first model, the red curve, represents the likelihood of cTHA including death rate and the statistical assumption that a deceased patient will not undergo subsequent cTHA. This model represents the baseline likelihood of cTHA, while the two other models incorporated additional factors. The green curve represents a scenario where patients would not die but continue to receive cTHA with the same likelihood as all survivors. The blue curve considers the likelihood of cTHA with censored death and the assumption that every deceased patient would have undergone subsequent cTHA. This model combines the baseline likelihood of the red curve, with addition of the percentage of deceased patients over time.

Secondary parameters such as age, gender, body mass index, American Society of Anaesthesiology (ASA) score and diagnosis leading to THA were also collected, if available.

## Results

Data were available from 21 studies, including a total of 1,456,071 patients who underwent iTHA and subsequently received 249,117 cTHA, at least 12 months later (Table 3). One study included only cases with avascular necrosis of the femoral head (AVNFH) as primary diagnosis (20). Data coverage of secondary parameters was insufficient, prohibiting further analysis.

**Table 1:** The following are the Medical Subject Headings (MeSH) and terms utilized in the PubMed search process for this study.

Search	Query	Items found	Explanation of the search
#9	((‘hip prosthesis’[MeSH terms] OR ‘arthroplasty, replacement, hip’[MeSH terms]) AND (‘contralateral*’[text word] OR ‘subsequent*’[text word] OR ‘bilateral*’[text word])) AND (humans[filter])	3,680	Search: (#6) AND (#7); filters: humans
#8	(‘Hip prosthesis’[MeSH terms] OR ‘arthroplasty, replacement, hip’[MeSH terms]) AND (‘contralateral*’[text word] OR ‘subsequent*’[text word] OR ‘bilateral*’[text word])	3,825	Search: (#6) AND (#7)
#7	‘contralateral*’[text word] OR ‘subsequent*’[text word] OR ‘bilateral*’[text word]	1,300,300	Search: ((#3) OR (#4)) OR (#5)
#6	‘Hip prosthesis’[MeSH terms] OR ‘arthroplasty, replacement, hip’[MeSH terms]	45,802	Search: (#1) OR (#2)
#5	‘bilateral*’[text word]	311,069	
#4	‘subsequent*’[text word]	930,810	
#3	‘contralateral*’[text word]	95,840	
#2	‘Arthroplasty, replacement, hip’[MeSH terms]	32,559	
#1	‘Hip prosthesis’[MeSH terms]	24,978	

**Table 2:** The following are the Medical Subject Headings (MeSH) and terms utilized in the Cochrane search process for this study.

Search	Query	Items found	Explanation of the search
#8	((MeSH descriptor: [hip prosthesis] explode all trees) OR (MeSH descriptor: [arthroplasty, replacement, hip] explode all trees)) AND (((contralateral):ti,ab,kw) OR ((bilateral):ti,ab,kw) OR ((subsequent):ti,ab,kw))	241	Search #3 AND #7
#7	((contralateral):ti,ab,kw) OR ((bilateral):ti,ab,kw) OR ((subsequent):ti,ab,kw)	66,373	Search #4 OR #5 OR #6
#6	(subsequent):ti,ab,kw	40,466	
#5	(bilateral):ti,ab,kw	20,682	
#4	(contralateral):ti,ab,kw	7,113	
#3	(MeSH descriptor: [hip prosthesis] explode all trees) OR (MeSH descriptor: [arthroplasty, replacement, hip] explode all trees)	2,771	Search #1 OR #2
#2	MeSH descriptor: [arthroplasty, replacement, hip] explode all trees	2,092	
#1	MeSH descriptor: [hip prosthesis] explode all trees	1,168	

**Table 3:** Summary of studies included.

Reference	Year published	Country	Observation	Mean FU, years	iTHA, *n*	cTHA, *n*
Amstutz *et al.* (12)	2016	USA	1998–2010	11; 8.5^†^	367	59
Cnudde *et al.* (49)	2018	Sweden	1999–2012	5.6^†^	133,654	32,077
Espinosa *et al.* (40)	2019	Sweden	1992–2014	8, 6^†^	177,834	39,676
Garland *et al.* (48)	2015	Sweden	1992–2012	N/A	189,276	40,558
Gazendam *et al.* (58)	2022	Canada	1999–2019	1*	8,273	1,213
Gillam *et al.* (38)	2012	Australia	2002–2008	N/A	84,759	9,997
Gillam *et al.* (46)	2013	Australia	2002–2010	N/A	78,634	12,668
Gillam *et al.* (46)	2013	Norway	2002–2010	N/A	19,786	3,867
Goker *et al.* (59)	2000	USA	1984–1985	8.7	99	21
Husted *et al.* (60)	1996	Denmark	1981–1994	5.6	1,199	356
Kim *et al.* (20)	2019	South Korea	2003–2011	7	121	59
Lamplot *et al.* (39)	2018	USA	2006–2013	>5	24,946	5,198
Lie *et al.* (18)	2004	Norway	1987–2000	5.3^†^	47,355	8,427
Moore *et al.* (61)	2022	USA	2010–2020	N/A	640,418	87,593
Ravi *et al.* (62)	2013	Canada	2002–2009	2	37,670	4,571
Ritter *et al.* (14)	1996	USA	1970–1980	N/A	179	96
Ritter *et al.* (14)	1996	USA	1970–1980	N/A	664	58
Sanders *et al.* (19)	2017	USA	1969–2008	12	1933	422
Santana *et al.* (17)	2020	USA	2004–2006	4	132	42
Sayeed *et al.* (13)	2009	USA	1969–1984	21.9	2,547	903
Sayeed *et al.* (41)	2012	USA	2001–2008	8.6	332	74
Shakoor *et al.* (37)	2002	USA	1981–2001	N/A	3,458	439
Shao *et al.* (63)	2013	China	1987–2000	17.8	2,435	743

N/A, data not available; FU, follow-up; iTHA, index THA; cTHA, contralateral THA.

* Follow-up data given in the text without connection to extracted data.

^†^ Values are median.

The overall likelihood of cTHA, considering death rate for the age group 65–69 years old in industrialized countries as a competing risk, was 17.8% (95% confidence interval 12.3–23.3%) within 5 years after iTHA and increased to 22.7% (16.1–29.4%) within 10 years, respectively (Fig. 2, red curve). At 15 and 20 years, this likelihood increased further to 25.6% (18.3–32.9%) and 27.7% (19.9–35.5%), respectively. Assuming deceased patients would continue to receive cTHA with the same likelihood as indicated above (red curve), the likelihood of cTHA increased to 18.1% (12.6–23.6%) after 5 years and 23.4% (16.8–30.1%) after 10 years, respectively (Fig. 2, green curve). The hypothetical scenario where every deceased patient would have undergone subsequent cTHA over time (blue curve) showed a likelihood of 19.7% (14.2–25.2%) within 5 years after iTHA and 25.7% (19.1–32.4%) after 10 years (Fig. 2, blue curve).

Patients undergoing iTHA due to AVNFH exhibited notably elevated likelihood of requiring cTHA of 49% within a mean of 7 years of follow-up (20).

## Discussion

To the best of our knowledge, this study is the first systematic review of the literature with meta-analysis performed to assess the likelihood of cTHA over time. Most of the cTHAs were required within the first years and nearly every fourth THA patient required cTHA within 10 years.

Considering the necessary duration of follow-up to evaluate the outcome, death must be considered as a competing risk. As patients die, they are no longer at risk for cTHA, which inherently lowers the observed rates of contralateral surgery. Ignoring this factor could lead to an overestimation of the likelihood of cTHA, especially in older populations, by definition more likely to suffer this outcome. Respectively, cTHA rates would be underestimated in younger populations exempt from significant mortality during the relevant follow-up. Therefore, mortality data from the global WHO database were integrated into the analysis. We chose the index mortality rate from the group 65–69 years of age, as this corresponds to the typical average age of THA patients (21, 22, 23, 24). Thus, patients are old enough to reflect the most common THA demographic, yet young enough that mortality does not overwhelmingly bias the results. However, it is important to recognize that the evaluation of death as a competing risk using the high-income group, as defined by the World Bank, is only an approximation and results may vary among countries. Nonetheless, our decision to focus on high-income countries aligns with the population represented in the studies included (Table 3).

Our basic model (Fig. 2, red curve) represents the likelihood of cTHA, assuming deceased patients would not undergo subsequent cTHA. As mortality risks may vary, this must be considered when counseling individual patients. It shows the minimal likelihood of cTHA over time, varying from 17.8% after 5 years to 22.7% after 10 years, which appears to fall within the lower third of previously published ranges (13–29% at 5 years after iTHA and 8.7–54% at 10 years after iTHA) (12, 13, 14). Most of the patients analyzed suffered OA as primary diagnosis, while the lifetime likelihood of requiring THA for OA in high-income countries is reported to range from 8.7 to 15.9% for females and from 6.3 to 8.6% for males (8), and while other diagnoses such as AVNFH may be associated with much higher rates of cTHA (20). The discrepancies between our findings and previous studies can be attributed to several factors. We defined iTHA and cTHA independently of the etiology. The variability in reported likelihoods may be due to differences in inclusion criteria not only linked to etiology, but also to age of the patients. Furthermore, different study designs and data extraction methods may also contribute to the varying reported outcomes.

The second model (Fig. 2, green curve) considers a scenario where patients would not die but continue to receive cTHA with the same likelihood as in the basic red model. This is particularly applicable to younger patients than the group aged 65 – 69 years considered for incorporation of the mortality risk. Although the difference between the minimal likelihood (Fig. 2, red curve) and the potential likelihood without mortality (Fig. 2, green curve) is small, it may also represent a realistic increase in cTHA due to rising life expectancy in the future, as life expectancies are calculated on current mortality data and thus suffer from a time lag.

The third model (Fig. 2, blue curve) is a hypothetical scenario, considering the likelihood of cTHA with censored death and the assumption that every deceased patient would have undergone subsequent cTHA over time. It represents the upper limit of potential cTHA occurrence in our analysis. This model presents a valuable framework for the assessment of younger patients with much lower mortality than the reference group incorporated into the analysis. The National Joint Registry of the UK showed that younger patients had lower risk of death at time of iTHA, with approximately half the risk of death for male patient below the age of 55, in comparison to their counterparts aged 65–69 years (25). Thus, they have more time to potentially undergo cTHA over the remaining lifespan. Analyzing a younger cohort with a lower risk of death in our study would likely result in the three curves aligning over time. This hypothetical convergence underlines the role of age as a pivotal determinant in this analysis. However, age distribution of elective primary iTHA varies, even if the mean age is similar among the various reports. The American Joint Replacement Registry (AJRR) reports 19.7% of patients being within the age of 50–59 years, with a mean age of 65.7 years, whereas the German Arthroplasty Registry (EPRD) reports 20.9% of patients aged 55–64 years, with a higher mean age of 72 years (23, 24). The Swiss National Hip and Knee Joint Registry (SIRIS) Report 2022 shows stable age groups since 2016, with 66.8% of THAs performed in patients older than 65 years of age (21). As delineated in the 2022 Annual Report of the Australian Orthopaedic Association National Joint Replacement Registry, an estimated 35% of patients undergoing THA within the period spanning from 2003 to 2021 were under the age of 65 (22).

Meanwhile, the incidence of primary THA is rising, varying in the Australian arthroplasty registry report from 86.6 per 100,000 inhabitants in 2003 to 160.9 in 2021, and from 208 per 100,000 inhabitants in 2013 to 250 in 2021 in the Swiss arthroplasty registry report (21, 22). THA projections for the United States show total numbers increasing by up to 174% until the year 2030 (9, 26). In Organization for Economic Co-operation and Development (OECD) countries, numbers of THA are expected to grow from 1.8 million per year in 2015 to 2.8 million in 2050 (15). Numbers of THA performed in Australia, New Zealand, Ireland, Norway and Switzerland are expected to double until 2050 (15). Despite these predictions, the crucial inquiry of the optimal postoperative follow-up strategy for these individuals remains unresolved, with a large variation in timing of postoperative follow-up examinations (27). Current guidelines recommend routine follow-up at 10 years after iTHA. However, there is significant variability in the number of intermediate examinations leading up to this 10-year timeframe. Recommendations range from the two to six examinations until the 10-year follow-up (27, 28, 29, 30, 31). Our findings suggest that the likelihood of requiring cTHA within the initial 10 years is approximately twice as high as the likelihood of requiring revision (32, 33, 34, 35, 36). Interestingly, despite this observation, the existing literature tends to focus on the latter aspect (32, 33, 34, 35, 36). One might consider more frequent follow-up examinations during the first 3–5 years to assess patient needs for cTHA and provide adequate counseling and treatment rather than basing the follow-up schedule primarily on the less probable need for revision of the iTHA. Waiting for these patients to spontaneously present themselves might be inadequate, especially among the elderly and cognitively impaired patients, considering the costs and the cognitive decline induced by OA (3, 7). Furthermore, it remains unclear whether a more satisfied patient presents for earlier cTHA than a less satisfied patient. With rising financial pressure on healthcare systems, our data may affirm proactive assessment for potential total joint arthroplasty (TJA) necessity (5).

Our analysis of likelihood of cTHA extended beyond patients with OA to encompass all etiologies. However, OA remains the most common diagnosis leading to THA and usually is not a process affecting solely a single joint (37, 38). In a large database study of over 85,000 patients who underwent THA, total knee arthroplasty (TKA) and total shoulder arthroplasty, 23% underwent contralateral arthroplasty of the same joint within 5–8 years after the index operation (39). Obesity was reported as the greatest risk factor (39). According to a recent analysis of the Swedish Hip and Knee Register (SHAR and SKAR), with over 300,000 patients included, the highest risk for subsequent TJA affects the same joint on the contralateral side (40). Therefore, it is important to consider the risk of contralateral joint deterioration and the potential need for future surgery when considering iTHA for patients with primary OA. Based on combined severity of clinical symptoms and radiographic findings, low-risk patients showed only 1% chance of contralateral progression of OA, whereas high-risk patients had a 97% chance of OA progression, resulting in cTHA (41). However, cohorts with cTHA due to less common primary diagnosis other than OA, e.g., AVNFH only, showed a particularly high risk of cTHA (20, 42), whereas patients with developmental dysplasia of the hip (DDH) have a higher probability of progressing to end-stage osteoarthritis or THA at 10- and 20-year follow-up compared with femoroacetabular impingement (FAI) and normal morphology (43). In contrast, patients undergoing periacetabular osteotomy (PAO) for hip dysplasia typically have lower rates of advanced osteoarthritis and are significantly younger at the time of surgery, which results in a lower and less comparable risk for cTHA (44). These patients show long-term survivorship of the treated hip joint, with a 30-year cumulative survival rate free from THA of 29%, a rate which may not automatically be transposed to the contralateral hip joint.

This study has some limitations. There were single institution studies and multicenter registry analysis, retrospective and prospective study designs, small studies and population or national registry reports with large sample sizes. Data had to be extracted from tables, figures and KM survivorship curves, the latter being a possible source of error. However, data extraction from Kaplan–Meier (KM) graphs was conducted using an automated method to limit measurement bias and reduce the inaccuracies often associated with manual data extraction. An inherent risk of this meta-analysis is the issue of selection bias, since the investigated groups cannot be equal with respect to primary diagnosis, distribution of ethnicities or access to medical care, to name only a few. The large sample size of this review, including data from over a million patients, minimizes the impact of outliers or smaller studies. However, all studies included were conducted in industrialized countries with presumably similar – or at least comparable – access to the healthcare system, which should enhance the generalizability of our findings (45). Although most arthroplasty registries do not routinely include data on comorbidities, it is essential to recognize their impact. Patients with significant comorbidities may face challenges for secondary operations, potentially affecting the observed likelihood of cTHA.

Furthermore, a small part of the studies documented the first and second TJA procedures, including both hips and knees (40, 46). Even though it was not possible to determine if later TJA involved cTHA or TKA, these studies were included in the analysis. This should have no relevant effect on our results, considering that overall, the outcomes of more than a million patients could be in our analysis. In addition, data coverage of the included hip arthroplasty registries, which represent the predominant part of the data analyzed, is high to almost complete (18, 38, 40, 46, 47, 48, 49). The possibility of overlapping data between different studies cannot be ruled out completely. However, this factor should be considered irrelevant in terms of the overall distribution of the included studies since there is no direct geographical overlap among them.

This study is strengthened by its design. Published studies have been identified through searches of two different databases. Three models have been constructed to consider the competing risk of death and the influence of age at iTHA on the likelihood of cTHA. However, KM analysis provides only an estimate, with a potential for deviation in case of competing risks, since the KM method treats those who died (having no more risk of cTHA) similarly to those who are lost to follow-up (who could still be subject to cTHA). Thus, KM findings may suffer from underestimation (50). The notable strength of the green curve model lies in its mitigation of this bias. It illustrates the scenario, where patients would not die but continue to receive cTHA with the same likelihood, as indicated in the red curve.

To determine the long-term likelihood over time of cTHA after iTHA more accurately, we arbitrarily did not include patients with cTHA within 12 months. This affected two studies (19, 39). Those patients most probably presented from the beginning with bilateral disease, leading to cTHA shortly after recovery from iTHA, not corresponding to the study question of requirement for later cTHA. Particularly, since there is no uniform definition for simultaneously or planned staged bilateral THA (48), misclassification and/or insufficient data documentation may otherwise have biased this analysis. The debate about the role of simultaneous bilateral THA (sbTHA) goes beyond the scope of this review (51, 52, 53, 54).

To the best of our knowledge, no other study has analyzed the risk of cTHA within the scope of a meta-analysis. The results suggest that every sixth patient receiving iTHA undergoes cTHA within 5 years and about every fourth patient within 10 years. Addressing patient concerns regarding the risk of cTHA may be relevant to improve patient satisfaction. Moreover, it may decrease healthcare burden, as OA reduces QoL, impairs cognitive function and conservative treatment also induces costs (1, 2, 3, 4, 5, 6, 7). The increasing incidence rate of primary THA worldwide emphasizes the importance of understanding and addressing the likelihood of cTHA (9, 15, 45, 55, 56, 57). This knowledge can help in implementing cost-effective strategies, optimizing THA follow-up, especially considering the higher likelihood of requiring a cTHA than the risk for revision of iTHA, according to our data (32, 33, 34, 35, 36). Conducting a prospective study to analyze the risk of cTHA within 3–12 months after iTHA would be important to refine the analysis. We therefore emphasize the necessity of further studies investigating the risk of cTHA.

## ICMJE Statement of Interest

The authors declare that there is no conflict of interest that could be perceived as prejudicing the impartiality of the work reported.

## Funding Statement

This research did not receive any specific grant from any funding agency in the public, commercial or not-for-profit sector.

## Author contribution statement

Conceptualization was done by CM, PW and JL. All authors helped in methodology. Formal analysis was done by MS. JL and MS helped in investigation. Writing of the original draft was done by JL. CM, PW and MS helped in review writing and editing. MS helped in visualization. Supervision was done by PW.
